# Dimensional Stability of Light-Activated Urethane Dimethacrylate Denture Base Resins

**DOI:** 10.3390/polym15030744

**Published:** 2023-02-01

**Authors:** Swati Mishra, Saurabh Chaturvedi, Mariyam Ali, Kaushik Kumar Pandey, Nasser M. Alqahtani, Mohammed A. Alfarsi, Mohamed Khaled Addas, Sunil Kumar Vaddamanu, Nasser M. Al Ahmari, Saeed M. Alqahtani, Ashfaq Yaqoob, Waleed M. S. Alqahtani

**Affiliations:** 1 Department of Prosthodontics, Career Postgraduate Institute of Dental Sciences, Lucknow 226008, India; 2 Department of Prosthetic Dentistry, College of Dentistry, King Khalid University, Abha 61421, Saudi Arabia; 3 Department of Dental Technology, College of Applied Medical Sciences, King Khalid University, Abha 61421, Saudi Arabia

**Keywords:** UDMA, record base, light cure denture base, dimensional discrepancy

## Abstract

An accurate and dimensionally stable trial denture base is required for a successful denture. The aim of this in vitro study was to assess the dimensional stability of a light-activated urethane dimethacrylate (UDMA) visible light cure (VLC) denture base with three fabrication techniques and different curing cycles. Forty-five VLC denture base samples were divided evenly into three groups. Group A used a conventional fabrication technique with a curing cycle of 5 min. Group B used a modified fabrication technique with two 4-min curing cycles. Group C used a multi-step fabrication technique with three curing cycles (4 min, plus 4 min, plus 2 min). The samples were sectioned and observed under a stereomicroscope to measure the discrepancy between the sample and the master cast. The mean dimensional discrepancy (mm) at the molar region at mid-palate, after 24 h in Group A, B and C was 0.790 mm, 0.741 mm and 0.379 mm, respectively; at the right ridge crest, it was 0.567, 0.408 and 0.185, while at the left ridge crest it was 0.475, 0.331 and 0.125, respectively. Statistical analysis showed significantly different dimensional discrepancies among the groups at all three sites; right ridge crest (F = 93.54, *p* < 0.001), left ridge crest (F = 105.96, *p* < 0.001) and mid-palate (F = 125.53, *p* < 0.001). Within the limitations of this laboratory study, it can be concluded that the denture base using a multi-step fabrication technique with three curing cycles provides better adaptation than the conventional technique. The significance of the study is that clinicians should consider performing denture base fabrication using a multi-step technique to enhance adaptation and hence the stability of the dentures for patients.

## 1. Introduction

An accurate and dimensionally stable trial denture base is mandatory for the construction of a successful denture. To achieve intraoral stability and retention of the record base, the base should maintain close adaptation to the cast and should be dimensionally stable. The adaptation of a record base, however, depends on many factors, including the materials and methods used for its construction. It is a proven fact that the more dimensionally accurate and stable a material is, the more retentive and stable the denture will be. In the past, various materials were used to fabricate custom trays, record bases and dentures such as Shellac, Thermoform, Polycaproilaitone, Self-Cure Acrylic Resin and Heat Cure Acrylic Resin. The problem with these acrylic resins is that they shrink and become discoloured over time due to the absorption of water and oral fluids. They also undergo significant volumetric and optical changes in the conditions of the oral environment [1,2,3,4,5].

A lack of dimensional stability is one of the major shortcomings of acrylic resins. These dimensional changes occur either in the form of shrinkage or expansion and are mainly affected by the method of curing, along with other factors. The polymerization shrinkage during curing and the stresses released during flask cooling in heat-cured acrylic resins result in changes in dimensions and their magnitude is mainly affected by acrylic resin thickness [6,7,8]. Other factors include polymerization techniques, in which the difference in thermal expansion of gypsum and acrylic resin results in the development of internal stresses and variation in base thickness at different sites inside the flask results in altered record base adaptation and stability [9,10,11,12]. On the other hand, few authors stated that there is a reduction in the molecular weight of the resulting polymer chains after curing and so the variation in the curing techniques may not alter significantly the pattern of dimensional changes in acrylic resin.

The changes in the dimensions of the acrylic resins may affect patient satisfaction and negatively impact the stability and retention of record bases and ultimately the success of the prosthesis [9]. In their study, Consani et al. showed that the molar region is the most consistent site to produce the gap between the palatal area and the acrylic record base. They reasoned that it represents a linear shrinkage which occurs in the acrylic resin [10].

Visible light cure denture has attracted special attention recently as a new record base material and has been used worldwide as an alternative to the chemical acrylic resin material since 1984. It was marketed under the trade name “triad” and was suitable for many prosthodontic applications, including removable, fixed and maxilla facial prostheses. Light-activated urethane dimethacrylate (UDMA) denture base polymers were developed to overcome the allergies, the pungent smell in the laboratory and the conventional time-consuming lost wax method of fabricating prosthesis Polymethyl methacrylate (PMMA) materials [12]. The VLC resin was promoted as a material containing a urethane dimethacrylate matrix with an acrylic copolymer, micro-tins, silica filler, and a photo-initiator system. Triad, as a brand. was the first system of VLC-UDMA denture base polymer. It was presented in the market as a biocompatible material which has ease of fabrication and manipulation, low bacterial adherence, and the ability to bond to other denture base resins [13,14,15,16].

Overall, in the literature, the various advantages documented for VLC acrylic resin include rapid preparation time, good rigidity and strength, uniform thickness, and ease of use. Conversely, the disadvantages mentioned are the requirement of a curing unit, which also increases expenditure, stickiness on the surface after curing, difficulty in trimming and poor finish [15,16,17,18]. Nevertheless, the main problem reported for VLC record bases is dimensional instability, which is not only affected by material properties and technique, but also by the anatomy of the structure over which the prosthesis is to be made.

One of the major factors affecting dimensional stability is processing shrinkage. The processing shrinkage of the record base is affected by the shape of the palatal concavity and thus shrinkage occurs toward the residual ridge, leading to the lifting of the record base in the mid-palatal region which was cited by Won-suck et al. [8]. The space beneath the posterior palatal region results from processing shrinkage. This dimensional change, that occurs during the denture processing, was recognized by several studies previously and the most possible explanation for it is that the strain release in the maxillary denture base tends to draw the flange inward and the resulting premature contact (gap) of the denture base with the mold in these regions causes the palate to be elevated. Previous research into VLC documented that the material close to the light cure unit would get polymerized first, resulting in gap formation due to transformation and contraction [19]. The cause for the gap formation with the master cast is the contraction because of polymerization and cooling, as well as being lifted by internal stress during polymerization [2,8,12,18]. VLC resins are considered to be dimensionally stable directly after complete polymerization with the use of a proper light source and exposure time [1,2,17,18].

Studies on light-curing resin demonstrated that the nearest material will polymerize first when exposed to polymerizing light, leading to the establishment of a gap due to transformation and contraction [16]. The contraction brought on by polymerization and cooling, as well as the lifting caused by internal stress during polymerization, are the causes of this gap’s creation with the master cast [16,17,18].

Consequently, recommendations have been made in previous studies for improving the accuracy of the VLC resin, whether by a multiple-step curing cycle or by dividing the VLC sheet into two parts along the junction of horizontal and vertical parts of the palate [16,20,21]. By these methods, the stresses that develop at the time of polymerization can be restricted to that segment of the material and shift the direction of shrinkage from the ridge crest area to two separate areas in the middle of the palate and the crest of the ridge [21,22,23].

Nevertheless, still discrepancies have been reported in the adaptation of visible light cure record bases, despite using different techniques. We decided to explore this area more and the goal set was to formulate an accurate method which can be used on a regular basis with ease to produce VLC record bases with better adaptation so as to accompanied successful prosthesis processes. Thus, this study was conducted with the aim to assess and compare the dimensional stability of light-activated urethane dimethacrylate (UDMA) visible light cure (VLC) denture base, fabricated by different techniques and different curing cycles. The objective was to decide and recommend a technique which gives the most stable record base for regular laboratory use and to determine whether time has any effect on the dimensional stability of VLC record bases.

## 2. Materials and Methods

The present cross-sectional study was conducted in the Department of Prosthodontics, Career Post Graduate Institute of Dental Sciences and Hospital, Lucknow, India. It was approved by the institute’s ethical committee, and the ethical waiver was obtained as there was no involvement of human subjects or tissues (SRC/CPGIDSH/ETHW–11–2019). This study was conducted with the aim of evaluating and comparing the dimensional stability of VLC denture bases fabricated by different techniques and different curing cycles. The objective was to decide on and recommend a technique which gives the most stable record base for regular laboratory use and to determine whether time has any effect on the dimensional stability of VLC record bases.

### 2.1. Sample Size Calculation

The sample size was selected based on a similar previous study in which the sample size was 30 [12]. Based on the recommendations for sample size calculations in in vitro studies, the number of samples should be in accordance with the statistical calculation of previous studies and should be 10–15% greater than the regular sample size in order to anticipate the incontinence in standards [13]. Thus, in the present study total sample size taken was 45, which was then divided into 3 groups of 15 samples each.

### 2.2. Fabrication of Samples

A completely edentulous patient was recorded in the outpatient department for denture fabrication. The oral examination was performed for a well-formed, rounded, completely healed edentulous residual alveolar ridge. Patient written consent was obtained before starting the study. The master cast was made after a secondary impression, following the conventional complete denture fabrication protocol.

**Figure 1:**
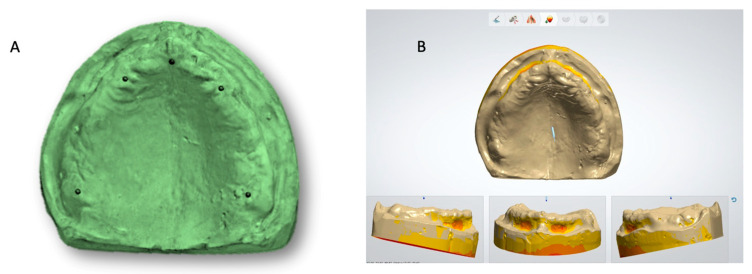
(**A**) Master cast for fabrication of metal master model; (**B**) Representative scanned images of master cast.

The master cast obtained in this way was scanned using a desktop laboratory scanner (D800, 3Shape A/S., Copenhagen, Denmark). The laboratory scanner software (2022.1; 3Shape A/S., Copenhagen, Denmark) has a self-alignment setting which joins the multiple scans of various sections and produces a complete virtual image. The data of the scan was stored in a standard tessellation language (STL) format for further use (Figure 1).

The stl file of the master cast was used in an advanced CAD-CAM software [(3-Shape Dental System–Complete Restorative–Complete Denture Module)-2022.1; 3Shape A/S, Copenhagen K Denmark] and printed in castable resin. With the help of a castable die, the metal master die was fabricated in metal [cobalt-chromium (Wirobond C; Bego Gmbh, Bremen, Germany)] (Figure 1). The metal master die so prepared was duplicated and study casts were poured with type IV die stone (GC Fujirock EP, Kortrijk, Belgium).

A total of 45 casts were made. For each cast, the VLC denture base was fabricated using 3 different techniques. Based on the technique used for the fabrication of denture bases, the samples were divided into 3 groups. 

Group A comprised 15 samples which used a conventional technique of fabrication with a short curing cycle of 5 min. 

Group B comprised 15 samples which used a new technique of fabrication with a two-step curing cycle (4 + 4 min). 

Group C comprised 15 samples which used a two-stage technique of fabrication with multiple steps in the curing cycle (4 + 4 + 2 min). 

All the samples were cut at a specific region and observed under a stereomicroscope. The gap/discrepancy between the master cast and VLC record bases at 3 different points was measured. Two times measurement was done after 12 h and 24 h after fabrication.

### 2.3. Making of Visible Light Cure Record Bases

The VLC record bases were fabricated over the maxillary casts. The VLC sheets were removed from the light protective packet and immediately adapted to the cast by hand pressure. A particular method was used for the adaptation of the VLC sheets. The adaptation started from the centre of the cast toward the periphery in order to reduce air bubbles. 

**Figure 2:**
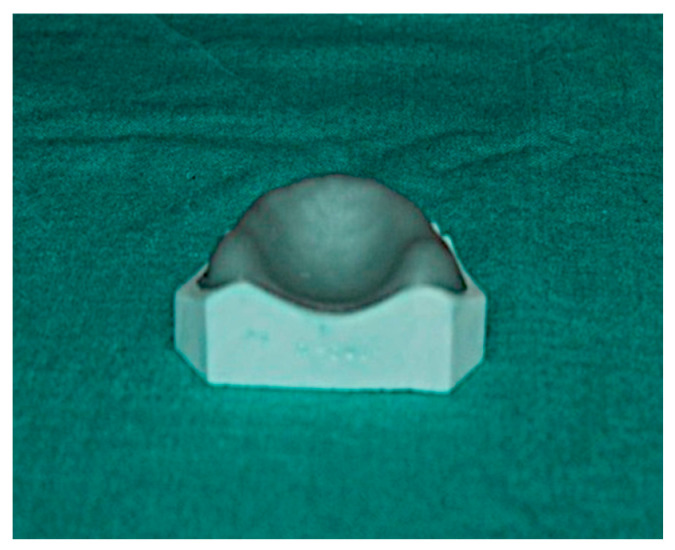
Record Base Fabricated by Conventional Technique (Group-A).

**Figure 3:**
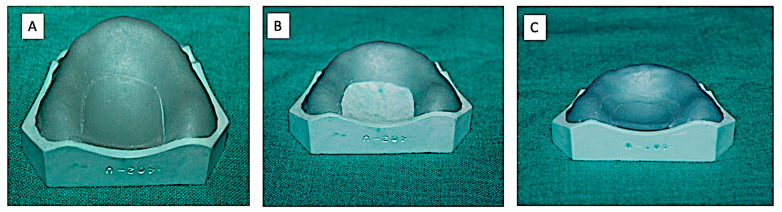
Record Base Fabricated by Technique-1 (Group-B). (**A**) The cut along the residual ridge 15 mm below the alveolar crest; (**B**) The remaining VLC sheet on the cast-cured for 4 min; (**C**) The cut U-shape segment replaced on the cast and cured for additional 4 min.

**Figure 4:**
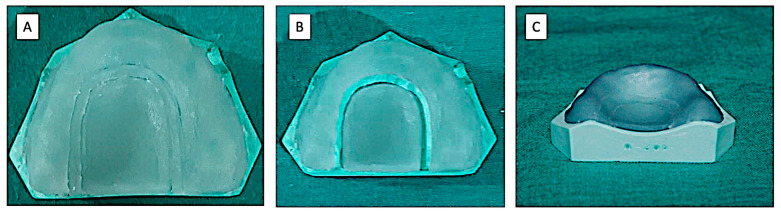
Record Base Fabricated by Technique-2 (Group-C). (**A**) The cut- below 15 mm from the alveolar ridge crest; (**B**) Fist components: a U-shaped segment along the residual ridge and (**C**) second component an elongated segment in the mid-palatal region.

Three techniques were used for making VLC record bases: 

(A) Conventional technique—Visible light cure sheets were adapted on the casts and excess material was cut off. Following this, the cast was placed along with the adapted VLC sheet in a light cure polymerizing unit (Tray-Lux) for 5 min (Figure 2). 

(B) Technique-1—Initially visible light cure sheets were adapted on the cast. Then, a U-shape segment of the adapted VLC sheet was cut along the residual ridge 15 mm below the alveolar crest. The remaining VLC sheet on the cast was then placed in the polymerizing unit for 4 min. The cast was removed from the unit and the cut U-shape segment was replaced on the cast and cured for additional 4 min (Figure 3). 

(C) Technique-2 (two-stage technique)—Visible light cure sheets were adapted on the cast. The excess material was cut from the border. Then, approximately 1.0 mm width of the VLC sheet material was cut at the junction of the horizontal and vertical plane of the palate. The cut was made below 15 mm from the alveolar ridge crest. This was done to obtain two separate components: a U-shaped segment along the residual ridge and an elongated segment in the mid-palatal region (Figure 4). The record base and cast assembly were placed in a polymerizing unit to cure for 4 min. After removing the assembly from the unit, the space between the two segments of the polymerizing sheet was filled using the cut-out VLC sheet. Then, the whole assembly was returned to the polymerizing unit for an additional 4 min. After removing from the unit, an air barrier coating was applied on the internal surface of the record base, and the whole assembly was returned to the polymerizing unit for an additional 2 min.

### 2.4. Sectioning of the Samples

**Figure 5:**
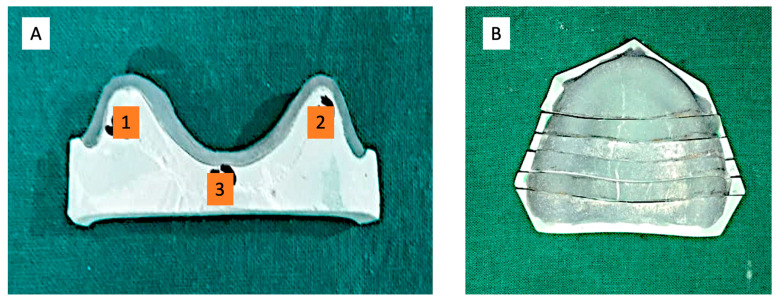
Sectioning & Marking of the Cast & Record Base Assembly (**A**) Coronal section view (1—right ridge crest; 2—left ridge crest and 3—mid-palate region); (**B**) Occlusal view.

The samples so prepared (cast and VLC record base assembly) were transversely sectioned with the help of a precision cutting machine (BUEHLER Worldwide, Waukegan Road Lake Bluff, IL, USA). The cuts were made at predetermined marked locations (canine region, molar region, posterior palatal seal region). In each region, 3 points (1- right ridge crest; 2- left ridge crest and 3- mid-palate region) were marked to measure the discrepancy:

The canine region (c), [right ridge crest (c1), left ridge crest (c2) and mid-palate region (c3)];
The molar region (m), [right ridge crest (m1), left ridge crest (m2), and mid-palatal region (m3)]
The posterior palatal seal region (p) [right ridge crest (p1), left ridge crest (p2), and posterior palatal seal region (p3)] (Figure 5).

### 2.5. Measuring the Discrepancy between Cast and Record Bases by Using a Stereomicroscope

**Figure 6:**
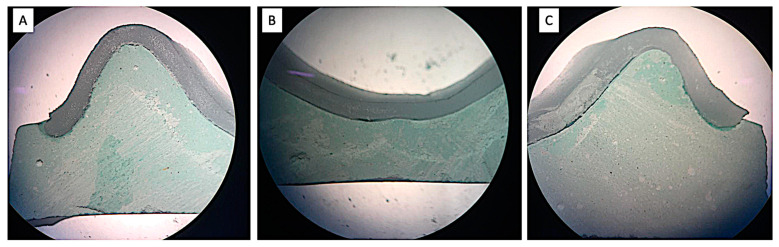
Discrepancy Seen Under the Stereomicroscope at (**A**) Right Ridge Crest; (**B**) Mid-Palate Region; (**C**) Left Ridge Crest.

After cutting all the samples, the discrepancy was measured at 9 pre-marked locations. The first measurement was performed after 12 h and the second after 24 h of record base fabrication. For evaluating the discrepancy, the perpendicular distances were measured from the internal surface of the record base to the external surface of the cast. Samples were studied under a stereomicroscope under a magnification of 20×, which can measure up to 0.001 mm (Figure 6). At each point, the discrepancy reading was recorded twice, and the average was taken as the final reading. All measurements were recorded by a trained technician who was blinded from the study. All measurements were recorded and analyzed using statistical analysis software (IBM SPSS Statistics for Windows v22; IBM Corp, Armonk, NY, USA).

### 2.6. Statistical Analysis

Microsoft Excel was used to calculate the means, standard deviation, and standard error of the mean (SEM). Two-way ANOVA, Tukey HSD and a *t* test was conducted to measure the statistical significance of differences among the groups. The Statistical Package for the Social Sciences (SPSS) software version 22 IBM.; Chicago (III., USA) was used to find the significant difference between the techniques. The significance level was set to 95%.

## 3. Results

The results of the study showed that there was a discrepancy between the record base and cast. The results were studied region- and time-wise.

### 3.1. Dimensional Discrepancy between Record Base and Cast after 12 h

The dimensional discrepancy of three groups (Group A, Group B and Group C) at three different sites (right ridge crest, left ridge crest and mid-palate) of the canine, region after 12 h was significantly different among the groups at both right ridge crest (F = 6.69, *p* = 0.003) and mid-palate (F = 10.34, *p* < 0.001), whereas the difference was insignificant at left ridge crest (F = 0.68, *p* = 0.512). For the molar region, there was a significantly different dimensional discrepancy among the groups at all three sites; right ridge crest (F = 40.01, *p* < 0.001), left ridge crest (F = 34.69, *p* < 0.001) and mid-palate (F = 67.81, *p* < 0.001). For the posterior palatal seal, the region showed significantly different dimensional discrepancies among the groups at all three sites; right ridge crest (F = 23.38, *p* < 0.001), left ridge crest (F = 23.27, *p* < 0.001) and mid-palate (F = 67.36, *p* < 0.001) (Table 1).

**Figure 7:**
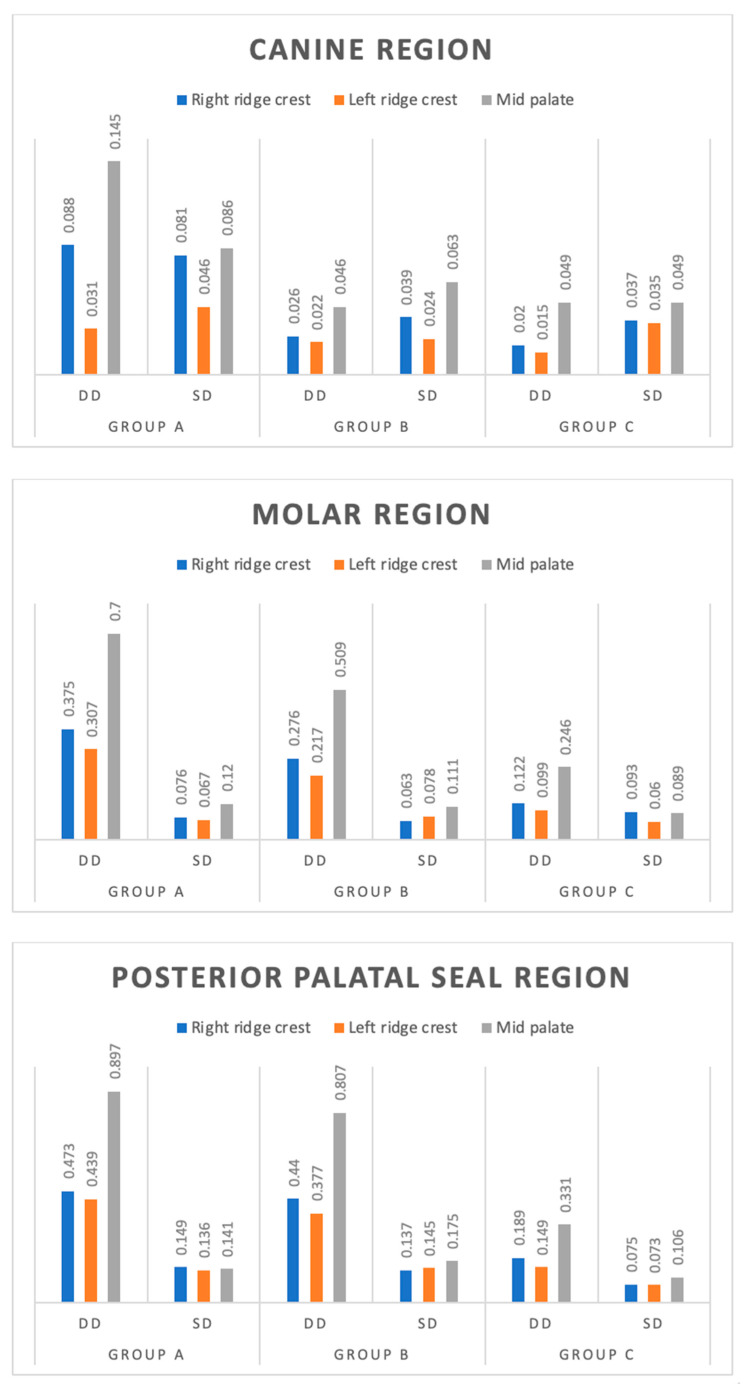
Mean dimensional discrepancy (mm) of three groups at three sites of canine, Molar and Posterior Palatal region at 12 h. Dimensional discrepancy (DD); Standard deviation (SD).

Further, for each site, comparing the difference in the mean dimensional discrepancy between the groups, the Tukey test showed a significantly (*p* < 0.05 or *p* < 0.01) different and lower dimensional discrepancy in both Group B and Group C as compared to Group A at all sites for canine and molar region. Furthermore, at all sites, it was also found to be significantly (*p* < 0.001) different and lower in Group C compared to Group B (Figure 7).

Tukey test showed significantly (*p* < 0.001) different and lower dimensional discrepancy in Group C, as compared to both Group A and Group B, at all sites. However, at all sites, it did not differ (*p* > 0.05) between Group A and Group B, i.e., found to be statistically the same (Figure 7).

### 3.2. Dimensional Discrepancy between Record Base and Cast after 24 h

The dimensional discrepancy of three groups (Group A, Group B and Group C) at three different sites (right ridge crest, left ridge crest and mid-palate) of the canine region after 24 h showed significantly different dimensional discrepancies among the groups at all three sites; right ridge crest (F = 71.42, *p* < 0.001), left ridge crest (F = 81.84, *p* < 0.001) and mid-palate (F = 59.21, *p* < 0.001). For the molar region, significantly different dimensional discrepancies among the groups at all three sites; right ridge crest (F = 93.54, *p* < 0.001), left ridge crest (F = 105.96, *p* < 0.001) and mid-palate (F = 125.53, *p* < 0.001). For the posterior palatal seal region, significantly different dimensional discrepancies among the groups at all three sites; right ridge crest (F = 96.97, *p* < 0.001), left ridge crest (F = 102.24, *p* < 0.001) and mid-palate (F = 14.56, *p* < 0.001).

**Figure 8:**
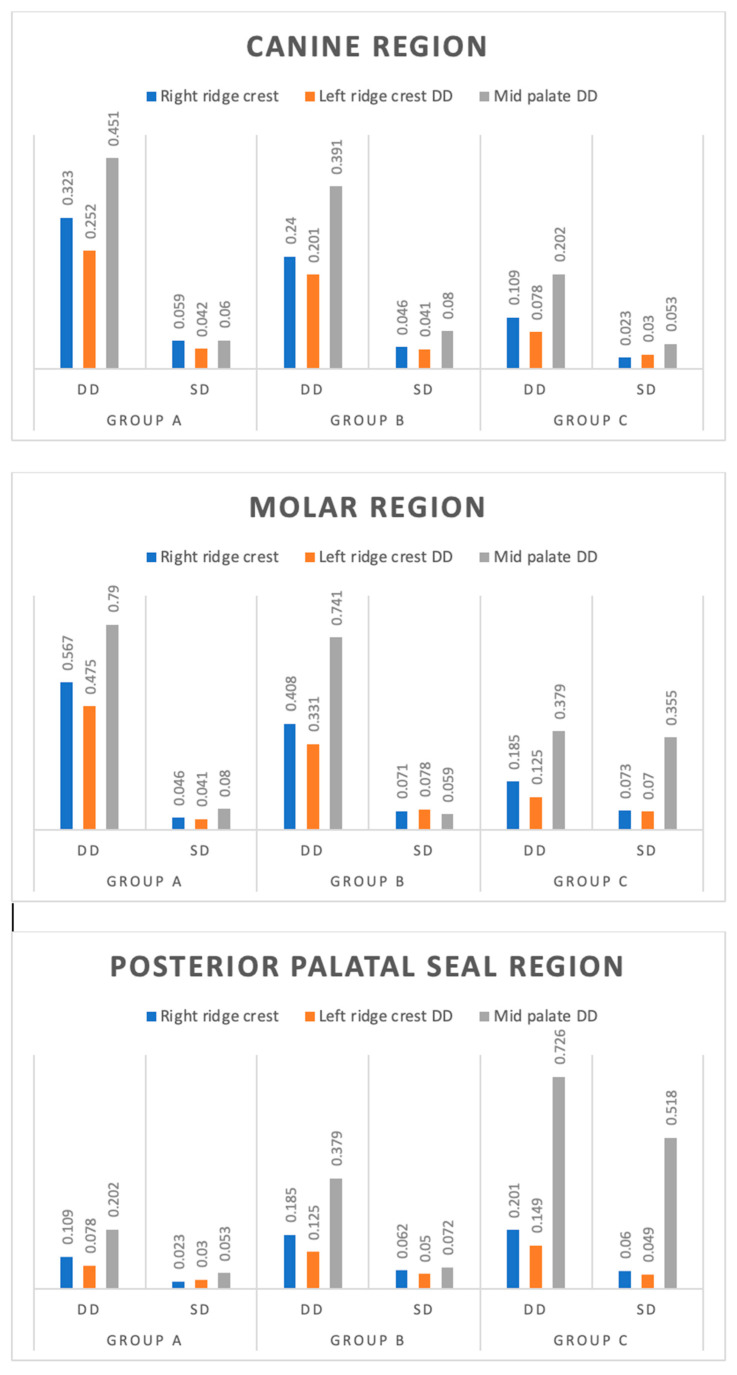
Mean dimensional discrepancy (mm) of three groups at three sites of canine, Molar and Posterior Palatal region at 24 h. Dimensional discrepancy (DD); Standard deviation (SD).

The Tukey test showed significantly (*p* < 0.05 or *p* < 0.01 or *p* < 0.001) different and lower dimensional discrepancies in both Group B and Group C as compared to Group A at all sites in both canine and molar regions (except Group A and Group B in mid-palate in the molar region.). Furthermore, at all sites, it also lowered significantly (*p* < 0.001) in Group C as compared to Group B. Moreover, in posterior palatal seal region, at both the right and left ridge crest, dimensional discrepancy also got lowered significantly (*p* < 0.001) in Group C as compared to Group B but at mid-palate, it did not differed (*p* > 0.05) between the two groups, i.e., found to be statistically the same (Table 2, Figure 8).

## 4. Discussion

Acrylic resins are widely used in dentistry to fabricate custom trays and record bases. These are used for making final impressions and recording maxillo-mandibular relations and arranging artificial teeth. To ensure intra-oral stability and the retention of the record base, the base should be closely adapted to the cast and should be dimensionally stable [18].

The introduction of auto-polymerizing acrylate polymers brought significant improvement to the mechanical properties of custom trays and record bases, but the use of self-cure resin has certain disadvantages such as shrinkage during polymerization, toxicity, the release of pungent vapors, residual monomer and adverse tissue reactions and related diseases. The improvement in the field of material science, especially in the field of dental materials, and the development of newer forms of denture base materials resulted in better denture base resins and helped in overcoming some of these drawbacks.

For example, the irritation to the mucosa and polymerization shrinkage is overwhelmed by polycarbonate, nylon and light-activated urethane dimethacrylate (UDMA) denture-base polymer. This VLC material is very common nowadays and is widely accepted both by patients and dentists because of its convenience to use, lack of monomers and superior dimensional stability after fabrication [24]. Additionally, its hazardous effects are very limited, the laboratory time required is significantly reduced time, and it has ease of use, good accuracy, strength and rigidity, uniform thickness, and good dimensional stability [25]. Visible light-cured resin is characterized by improved physical characteristics, such as increased stiffness, good form, volume stability, and low sensitivity to moisture. In addition, these materials are easy to use, and their use saves time [10,11,12]. Hence, the record base should be fabricated to be accurately fitted, and it should not change, but remain stable. Even the inaccurate record bases can look well fitted in the palate because of the elasticity of soft tissue, but it does not mean that it fits accurately in reality. The inaccurate record base incurs errors in the final occlusion of the complete denture due to the inaccurate jaw relation record [12,14]. The formation of the gap during the adaptation of the record base is very common in the posterior border of the maxillary cast. More specifically, it is formed in the posterior palatal seal area [21].

Different techniques have been suggested by various authors to overcome these defects and to obtain a more accurate fit. These techniques include the record base with elastic impression materials, vacuum adaptation of the record base, and staged polymerization by covering the palatal portion of the record base. However, the problem with resilient liners is tearing/detachment from the record base surface due to repeated insertion and removal, causing the inaccurate seating of the bases. The use of vacuum only improves adaptation, without ever addressing gap formation resulting from polymerization shrinkage [2].

The shrinkage of denture base acrylic resin is acknowledged as an error in the material; it results in space existing beneath the posterior palatal region. The most likely explanation for this dimension change, which has been noted in previous studies, is that the strain release in the maxillary denture base tends to draw the flange inward. As a result, there is an early contact (gap) of the denture base with the mold in these areas, which elevates the palate. These findings also show that acrylic resin’s inherent lack of dimensional precision is one of the potential causes of denture discrepancies [22,23,26,27,28].

Previous studies conducted comparisons of the self-cure and heat cure resins for adaptation using various techniques and determined that the pour technique is still a viable alternative for clinicians and dental technicians, as the fit of the denture is similar to that of the compression-moulding and injection-moulding techniques [19]. Even after three weeks of water storage, the residual monomer content of the heat-curing polymers is reduced but otherwise similar to that of the cold polymers [20]. These denture base acrylic resins will always contain an unreacted residual monomer that could irritate people and trigger allergies, regardless of the curing method used [22]. Heat-curing polymers also exhibit greater fracture resistance and less polymerization shrinkage, but they also exhibit higher stresses during the subsequent cooling phase.

The present study was conducted to evaluate and compare the dimensional discrepancy between cast and record bases using visible light cure material by different techniques and curing cycles at different time intervals. The results of the study showed that dimensional accuracy or fit of the denture base processed by technique 2, the two-stage technique with a multiple-step curing cycle, was better than that processed by the conventional curing cycle, and technique 1. Thus, the null hypothesis formulated was rejected. In the present study, the master metal model was made by using a castable model generated by 3-D printing, which helped in making uniform sample casts, thus eliminating the chance of error at the cast level.

Record bases, fabricated by a conventional method with a single-step curing cycle of 4 min showed more dimensional discrepancy due to the shape of the palatal concavity. Shrinkage occurred toward the residual ridge, leading to the lifting of the record base in the mid-palatal region, an occurrence which was cited by Won-suck et al. [2].

In techniques 1 and 2, i.e., with two steps curing cycle (4 min + 4 min) and a two-stage technique with a multiple-step curing cycle (4 min + 4 min + 2 min), the VLC sheet was cut along the residual ridge. Thus, the accuracy of the light-curing resin can be improved through multiple-step curing methods or by dividing the VLC sheet into two pieces along the junction of the horizontal and vertical configurations of the palate [21]. This method of cutting the VLC sheet into pieces and staged curing produced less discrepancy, which is a result of dividing the VLC resin sheets into two pieces along the palatal portion at a constant distance from the alveolar crest. By doing so, the distribution of contraction forces was performed evenly and applied stress to each of the pieces.

The edentulous maxilla consists of a relatively flat portion in the middle of the hard palate and inclined slopes towards the residual ridge. Due to the shape of palatal concavity, shrinkage occurs toward the residual ridge leading to the lifting of the record base in the mid-palatal region, as reported by Won-suck et al. [2].

The stresses that form during polymerization can be contained within each segment of the VLC sheet by cutting it into two pieces at the point where the palate’s horizontal and vertical configurations meet. This allows for the direction of shrinkage to be changed from the ridge crest area to two distinct areas in the middle part of the palate and at the crest. This study agrees with the study of Kenneth et al., in which it was described that the gap formation due to polymerization shrinkage could be reduced by limiting the amount of composite exposed to the curing light at one time and readjusting the uncured composite to the cast between curing cycles. Theoretically, compensation for discrepancies might be made for a record base, with a dimensional change of that proportion resting on compressible tissue without jeopardizing stability [21]. To achieve greater adaptability, it is advised that VLC record bases be cured using a two-stage approach with a multiple-step curing cycle based on the findings of this study.

In the present study, at the canine region, molar region, and posterior palatal seal region, the dimensional discrepancy was found to be the highest at the mid-palate region for all the groups (A, B, C) followed by the right ridge crest, and it was found to be lowest in left ridge crest. At the canine region among all the groups, a significant dimensional discrepancy was found at both the right ridge crest (c1) and mid-palate region (c3) and it was found to be insignificant at the left ridge crest (c2). Both Group B and Group C have shown a low dimensional discrepancy, as compared to Group A at both the right ridge crest (c1) and mid-palate (c3), which was significant. However, at the left ridge crest (c2) it was found to be similar between the groups after 12 h. The dimensional discrepancy was found to be similar at all sites (c1, c2, c3) between Group B and Group C after 12 h. These values are based on a previous study which is done by Kenneth G. Boberick et al. [21].

At the canine region, dimensional discrepancy was found significantly different (*p* < 0.001) at all three sites (c1, c2, c3) among all the groups after 24 h. In both Group B and Group C, the low and significant dimensional discrepancy was found at all sites as compared to Group A and the least dimensional discrepancy was found in Group C at all sites (c1, c2, c3) after 24 h.

At the molar region for all sites (m1, m2, m3), among all groups, a significant dimensional discrepancy was found (*p* < 0.001) after 12 h. A mid-palate region (m3) showed the highest discrepancy as compared to right ridge crests (m1) and left ridge crest (m2) among all groups. Due to the shape of the palatal concavity, shrinkage occurs toward the residual ridge, leading to the lifting of the record base in the mid-palatal region cited by Won-suck et al. [2].

Consani et al. suggested that the molar region is the most important site for gap space production between the palatal zone and the record base due to linear shrinkage [10]. At the molar region, in both Group B and Group C, the low dimensional discrepancy was found as compared to group A at all sites which were found significant (*p* < 0.001). This may be due to dividing the VLC sheet into two pieces along the junction of the horizontal and vertical configuration of the palate. The stresses that develop during polymerization can be confined within each segment of the material and lead to the direction of shrinkage shifting from the ridge crest area to two separate areas in the middle of the palate and the crest of the ridge [10].

At all sites, Group C was found to have the least dimensional discrepancy at the molar region as compared to Group A, and Group B was found to be significant after 12 h. This may be because of the light-polymerizing resin being divided into more pieces along the palatal portion by distributing force and limiting the amount of surface area exposed to the curing light and readapting the uncured visible light cure sheet to the cast between curing episodes [12]. At all the sites, in both Group B and Group C, mean dimensional discrepancy was found low and significant as compared to Group A after 24 h and it was found to be increased after 24 h as compared to the 12 h mark in the molar region.

At the posterior palatal seal region, for all sites (p1, p2, p3) dimensional discrepancy was found to be lower and significant in Group C as compared to Group A and Group B after 12 h. However, between Group A and Group B, it was found statistically insignificant after 12 h. For all sites (p1, p2, p3), dimensional discrepancy was found to be less significant in Group B and Group C as compared to Group A after 24 h and it was found to be more significant as compared to 12 h at all sites. In Group C at the mid-palate point, it was found statistically similar as compared to Group B after 24 h.

At the posterior palatal seal region, the dimensional discrepancy was found highest at all sites among all the regions. The greatest discrepancy occurred at the mid-palatal region of the posterior border, in the area of the posterior palatal seal, and gradually decreased anteriorly in agreement with the previous study [21]. The results of this study revealed a discrepancy pattern between the record base and cast, which is not uniform over the palatal surface [21].

In the present study, Group A showed the highest discrepancy as compared to Group B and Group C, at all the sites for all the regions Group B and Group C have shown low dimensional discrepancy of all the regions at a different site and it is found to be lowest with Group C after 12 and 24 h.

Therefore, it is recommended that the accuracy of the light-curing resin should be improved through multiple-step curing methods or by dividing the VLC sheet into two pieces along the junction of horizontal and vertical configurations of the palate. The completeness of polymerization is also a significant factor for two reasons: first, the degree of polymerization affects the geometric and material properties of the resultant prosthesis [29]. Secondly, the unreacted monomer may produce undesirable effects on the human body [30]. However, it has been reported in the literature that polymerized UDMA denture bases are nontoxic and that unpolymerized VLC materials have very low toxicity [31]. UDMA material has been proven to be less allergenic than other acrylics [32,33,34]. The recommendations for VLC denture bases by various authors are not only based on satisfactory strength and dimensional stability but also on ease of manipulation and fabrication [31,32,33,34,35,36].

Even though the study was conducted in a precise way, there are certain limitations to its usability, which include the limited total sample size. The cutting of cast and incorporation of dust created problems while determining the discrepancies. It is recommended to use a new technique of micro-computer tomography for the evaluation of the gap below the record base without cutting the samples. The effect of light absorption from the cast, which was green in colour, was not taken into consideration as it was reported in a previous study [16] that the dimensional accuracy of the VLC trial denture base could be improved by using a dark-coloured cast. Additionally, comparisons with the newer advanced CAD-CAM milled and 3D printed material was not done, which is recommended for future studies.

## 5. Conclusions

Within the limitations of the study, the following conclusion can be drawn: the most accurate light-activated urethane dimethacrylate (UDMA) visible light cure (VLC) denture base can be obtained by a two-stage technique with multiple steps curing cycles (as in Group C). The dimensional discrepancy increases with time, irrespective of the techniques used for base fabrication (record bases were found to be dimensionally unstable after 24 h).

## Figures and Tables

**Table 1:** For each site of canine region, comparisons of difference in mean dimensional discrepancy (mm) between groups by Tukey test at 12 h.

Anatomical Regions	Comparison	Right Ridge Crest		Left Ridge Crest		Mid-Palate	
		Mean Diff.	*p* Value	Mean Diff.	*p* Value	Mean Diff.	*p* Value
Canine region	Group A vs. Group B	0.062	0.012	0.009	0.789	0.099	0.001
	Group A vs. Group C	0.068	0.006	0.015	0.481	0.096	0.001
	Group B vs. Group C	0.006	0.954	0.007	0.869	0.003	0.990
Molar region	Group A vs. Group B	0.099	0.003	0.090	0.002	0.191	<0.001
	Group A vs. Group C	0.253	<0.001	0.208	<0.001	0.454	<0.001
	Group B vs. Group C	0.154	<0.001	0.118	<0.001	0.263	<0.001
Posterior palatal region	Group A vs. Group B	0.033	0.745	0.061	0.365	0.090	0.211
	Group A vs. Group C	0.284	<0.001	0.289	<0.001	0.566	<0.001
	Group B vs. Group C	0.251	<0.001	0.228	<0.001	0.476	<0.001

**Table 2:** For each site of the canine region, comparisons of difference in mean dimensional discrepancy (mm) between groups by Tukey test at 24 h.

Anatomical Regions	Comparison	Right Ridge Crest		Left Ridge Crest		Mid-Palate	
		Mean Diff.	*p* Value	Mean Diff.	*p* Value	Mean Diff.	*p* Value
Canine region	Group A vs. Group B	0.075	<0.001	0.051	0.002	0.060	0.042
	Group A vs. Group C	0.206	<0.001	0.174	<0.001	0.249	<0.001
	Group B vs. Group C	0.131	<0.001	0.123	<0.001	0.189	<0.001
Molar region	Group A vs. Group B	0.159	<0.001	0.145	<0.001	0.049	0.210
	Group A vs. Group C	0.381	<0.001	0.351	<0.001	0.411	<0.001
	Group B vs. Group C	0.223	<0.001	0.206	<0.001	0.362	<0.001
Posterior palatal region	Group A vs. Group B	0.133	<0.001	0.130	<0.001	0.805	0.003
	Group A vs. Group C	0.427	<0.001	0.381	<0.001	1.227	<0.001
	Group B vs. Group C	0.294	<0.001	0.251	<0.001	0.422	0.173

## Data Availability

Data can be made available on demand by the chief researcher for academic purposes by email.

## References

[1] Zissis A., Huggett R., Harrison A. (1991). Measurement method used for the determination of dimensional accuracy and stability of denture base material. J. Dent..

[2] Oh W.-S., May K.B. (2008). Two stage technique for optimum fit and stability of light-polymerized record bases. J. Prosthet. Dent..

[3] Chaturvedi S., Addas M.K., Alqahtani N.M., Al Ahmari N.M., Alfarsi M.A. (2021). Computerized occlusal forces analysis in complete dentures fabricated by additive and subtractive techniques. Technol. Health Care.

[4] Bonatti M.R., Cunha T.R., Regis R.R., Silva-Lovato C.H., Paranhos H.D., de Souza R.F. (2009). The effect of polymerization cycles on color stability of microwave-processed denture base resin. J. Prosthodont..

[5] Dimitrova M., Corsalini M., Kazakova R., Vlahova A., Chuchulska B., Barile G., Capodiferro S., Kazakov S. (2022). Comparison between Conventional PMMA and 3D Printed Resins for Denture Bases: A Narrative Review. J. Compos. Sci..

[6] Wong D., Cheng L.Y., Chow T.W., Clark R.K. (1999). Effect of processing method on the dimensional accuracy and water sorption of acrylic resin denture. J. Prosthet. Dent..

[7] Chaturvedi S., Addas M.K., Alqahtani N.M., Al Ahmari N.M., Alfarsi M.A. (2021). Clinical analysis of CAD-CAM milled and printed complete dentures using computerized occlusal force analyser. Technol. Health Care.

[8] Bartoloni J.A., Murchison D.F., Wofford D.T., Sarkar N.N. (2000). Degree of conversion in the denture base materials for varied polymerization techniques. J. Oral Rehabil..

[9] Crag R.G., Powers G.M. (2002). Restoration Dental Material.

[10] Consani R.L.X., Domitti S.S., Rizzatti Barbosa C.M., Consani S. (2002). Effect of commercial acrylic resin on dimensional accuracy of the maxillary denture base. Braz. Dent. J..

[11] Consani R.L.X., Paula A.B., Fugolin A.P.P., Pfeifer C.S. (2020). Strategies for Potential Toughening of Acrylic Denture Bases Polymerized with Microwave Energy. Braz. Dent. J..

[12] Fatihallah A.A., Mohamed M.R., Zia K.G. (2009). Evaluation of dimensional stability for denture bases in different curing techniques. Mustansiria Dent. J..

[13] Chander N.G. (2016). Standardization of in vitro studies. J. Indian Prosthodont. Soc..

[14] Diaz-Arnold A.M., Vargas M.A., Shaull K.L., Laffoon J.E., Qian F. (2008). Flexural and fatigue strengths of denture base resin. J. Prosthet. Dent..

[15] Elkholy S. (2016). Improving Dimensional Stability of Visible Light Cure Trail Denture Base using Different Colored Cast. J. Contemp. Dent..

[16] Huh B.J., Kang G.M., Shin W.S., Ryu J.J. (2010). Effect of two-phase fabrication method for the optimum fit of light-polymerized record bases. J. Adv. Prosthodont..

[17] Anulekha K., Parameshwari G., Taruna M., Tella S., Reddy R., Jagani A.S. (2016). A Comparative Evaluation of the Denture Base Accuracy along the Posterior Palatal Seal using Different Anchorage Systems in Complete Dentures. J. Adv. Med. Dent. Sci. Res..

[18] Choi H.J., Lim J.H., Jo I.H. (2000). A study on the accuracy of the record base of the complete denture to the master cast according to kinds of resin and polymerization method. J. Korean Acad. Prosthodont..

[19] Wirz J., Jaeger K., Schmidli F. (1990). Light-polymerized materials for custom impression trays. Int. J. Prosthodont..

[20] Andreopoulos A.G., Polyzois G.L. (1994). Repair of denture base resins using visible light-cured materials. J. Prosthet. Dent..

[21] Boberick K.G., McCool J. (1998). Dimensional stability of record bases fabricated from light-polymerized composite using two methods. J. Prosthet. Dent..

[22] Faot F., da Silva W.J., da Rosa R.S., Del Bel Cury A.A., Garcia R.C. (2009). Strength of denture base resins repaired with auto- and visible light-polymerized materials. J. Prosthodont..

[23] Pronych G.J., Sutow E.J., Sykora O. (2003). dimensional stability and dehydration of a thermoplastic polycarbonate based and two PMMA based denture resins. J. Oral Rehabil..

[24] Kazanji M., Majeed A. (2015). The effect of storage and curing time on dimensional changes of visible light cured acrylic denture base (VLCADB). IOSR J. Dent. Med. Sci..

[25] Elkholy S. (2011). Evaluation of dimensional stability of visible light cured custom tray for complete denture. Egypt. Dent. J..

[26] Takamata T., Setcos J.C., Philips R.W., Boone M.E. (1989). Adaptation of acrylic resin dentures as influenced by the activation mode of polymerization. J. Am. Dent. Assoc..

[27] Al-Mulla M.A.S., Huggett R., Brooks S.C., Murphy W.M. (1988). Some physical and mechanical properties of a visible light-activated material. Dent. Mater..

[28] Danesha G., Lippolda C., Mischke K. (2006). Polymerization characteristics of light- and auto-curing resins for individual splints. Dent. Mater..

[29] Gorman C.M., O’Sullivan M. (2006). Fabrication of a duplicate denture using visible light-polymerized resin as an interim denture base. J. Prosthet. Dent..

[30] Rode S.M., Cavalcanti B.N., Ferrielo V., Faria M.R., Villa N. (2007). Biocompatibility of Two Types of resins for Prosthetic Usage. Cienc. Odontol. Bras..

[31] Aoyama Y., Maeda M., Yokoi R., Saitoh A., Tsukimura N., Shimada A., Satoh T., Hayakawa J., Takeda T., Ishigami K. (1991). Basic studies on visible light-curing resin for denture base. Part 8. The hardening depth of denture relining material after penetration of light through the denture base resin. J. Nihon Univ. Sch. Dent..

[32] Elahi J.M., Abdullah M.A. (1994). Effect of different polymerization techniques on dimensional stability of record bases. J. Prosthet. Dent..

[33] Alkurt M., Yeşil Duymuş Z., Gundogdu M. (2014). Effect of repair resin type and surface treatment on the repair strength of heat-polymerized denture base resin. J. Prosthet. Dent..

[34] Khan S.B., Geerts G. (2009). Determining the dimensional stability, fracture toughness and flexural strength of light-cured acrylic resin custom tray material. Eur. J. Prosthodont. Restor. Dent..

[35] Chaturvedi S., Khaled A.M., Al Humaidi A.S.A., Al Qahtani A.M., Al Qahtani M.D. (2017). A Novel Approach to Determine the Prevalence of Type of Soft Palate Using Digital Intraoral Impression. Int. J. Dent..

[36] Chung K.H., Wadhwani C., Cagna D.R. (2014). A simple technique to control the heat generated during light polymerization of custom impression trays. J. Prosthet. Dent..

